# Chemical and Hormonal Effects on STAT5b-Dependent Sexual Dimorphism of the Liver Transcriptome

**DOI:** 10.1371/journal.pone.0150284

**Published:** 2016-03-09

**Authors:** Keiyu Oshida, David J. Waxman, J. Christopher Corton

**Affiliations:** 1 Integrated Systems Toxicology Division, National Health and Environmental Effects Research Laboratory/ Office of Research and Development, United States Environmental Protection Agency, Research Triangle Park, North Carolina, United States of America; 2 Division of Cell and Molecular Biology, Department of Biology and Bioinformatics Program, Boston University, Boston, MA 02215, United States of America; University of Nebraska Medical Center, UNITED STATES

## Abstract

The growth hormone (GH)-activated transcription factor signal transducer and activator of transcription 5b (STAT5b) is a key regulator of sexually dimorphic gene expression in the liver. Suppression of hepatic STAT5b signaling is associated with lipid metabolic dysfunction leading to steatosis and liver cancer. In the companion publication, a STAT5b biomarker gene set was identified and used in a rank-based test to predict both increases and decreases in liver STAT5b activation status/function with high (≥ 97%) accuracy. Here, this computational approach was used to identify chemicals and hormones that activate (masculinize) or suppress (feminize) STAT5b function in a large, annotated mouse liver and primary hepatocyte gene expression compendium. Exposure to dihydrotestosterone and thyroid hormone caused liver masculinization, whereas glucocorticoids, fibroblast growth factor 15, and angiotensin II caused liver feminization. In mouse models of diabetes and obesity, liver feminization was consistently observed and was at least partially reversed by leptin or resveratrol exposure. Chemical-induced feminization of male mouse liver gene expression profiles was a relatively frequent phenomenon: of 156 gene expression biosets from chemically-treated male mice, 29% showed feminization of liver STAT5b function, while <1% showed masculinization. Most (93%) of the biosets that exhibited feminization of male liver were also associated with activation of one or more xenobiotic-responsive receptors, most commonly constitutive activated receptor (CAR) or peroxisome proliferator-activated receptor alpha (PPARα). Feminization was consistently associated with increased expression of peroxisome proliferator-activated receptor gamma (*Pparg)* but not other lipogenic transcription factors linked to steatosis. GH-activated STAT5b signaling in mouse liver is thus commonly altered by diverse chemicals, and provides a linkage between chemical exposure and dysregulated gene expression associated with adverse effects on the liver.

## Introduction

Adverse outcome pathways are defined as a series of key, mechanistically-linked events, starting with a molecular initiating event in which a chemical or other stressor interacts with a molecular target proceeding to an adverse outcome in a tissue. Agencies such as the Environmental Protection Agency that regulate chemicals have ongoing efforts to define adverse outcome pathways and develop procedures for their prediction [1, 2]. Several adverse outcomes are associated with alteration in the activity of the transcription factor signal transducer and activator of transcription 5b (STAT5b). STAT5b, like the other six mammalian STAT family members, responds to a variety of extracellular cytokine and growth factor signals, notably growth hormone (GH) [3–5]. GH secretion by the pituitary gland is regulated by two opposing peptide hormones, GH-releasing hormone (GHRH or somatocrinin) and GH-inhibiting hormone (GHIH or somatostatin), which are both secreted by neurosecretory nuclei cells of the hypothalamus into venous blood surrounding the pituitary. The balance of these two peptides determines the temporal pattern of GH secretion by the pituitary gland and is affected by physiological stimulatory factors (e.g., exercise, nutrition, sleep) and inhibitory factors (e.g., free fatty acids) [6]. In rodentia and in a less pronounced manner in humans, the pattern of GH secretion from the anterior pituitary differs between sexes. Male rats and male mice have a highly pulsatile secretion pattern, which results in plasma GH peaks at regular intervals, each followed by a well defined GH-free interval. In females, pituitary GH secretion is more frequent, such that GH is present in plasma in a nearly continuous manner. These sex-dependent plasma GH profiles have a profound effect on the regulation of the liver transcriptome, affecting a large number of genes involved in xenobiotic metabolism and transport (for review, see Waxman and O’Connor, 2006 [7]). Thus, the hypothalamic-pituitary-liver (HPL) axis controlled by GH is an important determinant for many sex-dependent liver functions.

STAT5b is activated by the GH receptor-associated tyrosine kinase, Janus Kinase 2 (JAK2), which catalyzes STAT5b tyrosine phosphorylation, followed by STAT5b dimerization, translocation to the nucleus, and binding to DNA response elements in genes regulated by STAT5b. In male rat and mouse liver, STAT5b is directly activated in response to each incoming plasma GH pulse, whereas in female rat liver, the persistence of plasma GH stimulation leads to a partial desensitization of the STAT5b signaling pathway and substantially lower nuclear STAT5b protein than the peak levels seen in male liver [8–11]. Studies of STAT5b-deficient mice reveal that STAT5b is an essential mediator of the sex-dependent effects of GH on liver gene expression, with STAT5b-deficient male mice displaying feminization of the liver gene expression [12, 13], as well as reduced body growth rate at puberty [14, 15]. STAT5a, a minor liver STAT5 form that is >90% identical to STAT5b, also responds to sexually dimorphic plasma GH stimulation but is unable to compensate for the loss of STAT5b and the associated loss of sex-specific liver gene expression. In contrast to the widespread effects of the loss of STAT5b, STAT5a deficiency has only a limited effect on female-predominant genes, ~15% of which show decreased expression in STAT5a-deficient female liver [16].

Loss of GH-activated liver STAT5b activity leads to metabolic disturbances linked to obesity as well as fatty liver disease [17], the most common liver disease in humans. Hepatic steatosis can progress to an inflammatory state (steatohepatitis), sometimes leading to cirrhosis and hepatocellular carcinoma [18]. Fatty liver disease occurs in people with excess alcohol consumption (alcoholic fatty liver disease) and in people who are obese, either with or without insulin resistance (nonalcoholic steatohepatitis (NASH)) [19]. There is evidence for a higher incidence of nonalcoholic fatty liver disease in men although the basis for the bias is not known [20]. GH deficiency is clinically associated with a high incidence of NASH, which can be reversed by GH administration [21]. Similarly, mice with defects in liver GH signaling are susceptible to NASH. Deficiency in mouse GH caused by overexpression of human GH in the hypothalamus and pituitaries in transgenic mice results in liver steatosis and increases in peroxisome proliferator-activated receptor γ (*Pparg*) expression [22]. Mice carrying GH receptor (*Ghr*) genes in which the cytoplasmic JAK2-binding domain is truncated or deleted exhibit high fat diet-induced steatosis. These mice also develop a number of diet-independent effects upon aging, including steatosis, obesity and subcutaneous adipocyte hypertrophy [23]. Liver-specific Jak2-null mice have markedly elevated levels of GH, liver triglycerides, and plasma free fatty acids. The steatosis induced in these mice is likely due in part to induction of the fatty acid transporter *Cd36* by peroxisome proliferator-activated receptor γ (PPARγ), as treatment with a PPARγ antagonist reduces the expression of liver *Cd36* and decreases liver triglyceride content [24]. Mice in which the genetic locus encoding *Stat5b* and *Stat5a* (called *Stat5a/b*) was deleted in hepatocytes develop fatty livers and display impaired proliferation of hepatocytes following partial hepatectomy [17]. Loss of *Stat5a/b* leads to reduced expression of insulin-like growth factor 1 (IGF-1), a feedback inhibitory regulator of GH secretion, resulting in compensatory increases in circulating GH levels, insulin resistance, and increased insulin levels [17]. Hepatocyte-specific *STAT5a/b*-null mice develop steatosis, which in aged mice (68 weeks) progresses to liver tumors [25]. The increased steatosis in these mice may be related to the increased expression and activation of prolipogenic sterol regulatory element binding protein 1 (*Srebp1*) and increased *Pparg* expression [26]. Aging male STAT5b-null mice on a normal diet also become obese later in life (Udy et al., 1997). A mutation in the *STAT5B* gene in a mature human was also associated with striking obesity [27]. Thus, the functional impairment of STAT5b could be a molecular initiating event that leads to several adverse outcomes, including obesity, fatty liver, and liver cancer. A number of lipogenic regulators likely mediate these effects, but their linkage to STAT5b impairment is not well understood.

Assessment of chemical toxicity is traditionally carried out using exposure tests in animals. Given the fact that these tests are expensive, evaluate only one chemical at a time, and require large numbers of animals for evaluation, a 2007 National Research Council report “Toxicity testing in the 21^st^ Century” (NRC, 2007) recommended the eventual reduction or replacement of testing in animals with computationally-derived predictions based, in part, on the results of high-throughput screens performed in cell lines. In particular, a major effort of the EPA ToxCast and Tox21 chemical screening programs [28, 29] is to identify chemicals that disrupt normal homeostasis of hormone-regulated systems, i.e., chemicals that are “endocrine disruptors”. A major challenge is how to implement a screening strategy that incorporates the complexities of hormone regulatory systems and recognizes the potential for chemicals to interact with and disrupt these systems at different levels of biological organization.

Building on the work described in our companion study [30], we implement here a computationally-based screen to identify chemicals and hormones that alter the HPL-GH axis by assessment of changes in liver STAT5b function. The approach used employs a gene expression biomarker comprised of STAT5b-dependent genes and a statistical test for similarity called the Running Fisher test, which together were found to be very accurate in predicting STAT5b activation or suppression. These methods were used to examine a compendium of microarray datasets derived from livers of chemically- or hormonally-treated mice, mouse primary hepatocytes, and mouse hepatocyte-derived cell lines. Remarkably, a large number of chemicals that activate xenobiotic-responsive receptors were found to suppress liver STAT5b function, possibly through disruption of the HPL-GH axis. Given the relationships between suppression of hepatic STAT5b function and disruption of lipid homeostasis, we hypothesize that a subset of these chemicals cause effects in the liver, including fatty liver disease and hepatocarcinogenesis, in part through the suppression of STAT5b function.

## Methods

### Overall computational strategy

A bioset is a list of genes with fold-change values indicating differential expressed between two states, e.g., males vs. females or chemically-treated vs. vehicle control-treated, and can be obtained by genome-wide transcriptional profiling comparisons between two biological states, e.g., using microarrays. A detailed description of the methods used to screen a large gene expression compendium to identify biosets that are significantly associated with activation or suppression of liver STAT5b function are found in the Methods section of the companion paper [30]. Briefly, a screen for STAT5b modulators was carried out using a gene expression biomarker comprised of STAT5b-dependent genes showing sex-biased expression [30] and a statistical test of similarity to individual biosets in an annotated database of gene expression profiles of statistically filtered genes. The STAT5b biomarker gene set is a list of 144 genes that require STAT5b for sex differences in expression mouse liver, together with associated fold-change values, which correspond to average differences in expression between male and female mouse liver across multiple studies. The STAT5b biomarker was used to find biosets that exhibit positive or negative correlation between genes that overlap with the STAT5b biomarker in a commercially available gene expression database provided by NextBio (www.nextbio.com). The database contains over 117,000 lists of statistically filtered genes from over 17,500 microarray studies carried out in 16 species (as of January, 2015). The statistical algorithm used for the comparison (the Running Fisher algorithm) uses a fold-change rank-based statistic that provides for an assessment of the overlap in regulated genes and whether those overlapping genes are regulated in a similar manner, or in an opposite manner to the STAT5b biomarker gene set. Only biosets from mouse liver, mouse primary hepatocytes and hepatocyte-derived cell lines were evaluated in this study (~1,850 biosets all together). Biosets in the database were annotated externally for pertinent information about the study including the category of factor (e.g., hormone) and the specific name of the factor (e.g., testosterone) examined, facilitating comparisons across biosets. After comparison to the STAT5b biomarker, the Running Fisher algorithm generates a p-value and correlation direction for each bioset. Results are used to populate a master table containing the annotated information for each comparison. We previously used this strategy to identify factors that activate or suppress other transcription factors (AhR, CAR and PPARα) [31–33]. This approach, in which multiple comparisons of the same factor can be made, allows a weight of evidence evaluation for effects of each factor represented in the compendium on liver STAT5b function and avoids conclusions based on a small number of comparisons.

### Identification of differentially expressed genes in NextBio microarray datasets

A description of the criteria used to identify experiments suitable for analysis, and details of the microarray processing steps in the NextBio pipeline are presented in Oshida et al. (2015)[30] and are discussed in greater detail in Kupershmidt et al. (2010)[34].

### Identification of differentially expressed genes in an external database

Independent of the NextBio database, a database of gene expression changes was assembled by our group using comparisons from mouse liver, mouse primary hepatocytes and hepatocyte-derived cell lines. All of these experiments used Affymetrix microarrays. Details regarding the methods are found in Oshida et al. (2015)[30]. Briefly, cel files from the Affymetrix experiments were first analyzed by Bioconductor SimpleAffy [35] to assess sample quality followed by normalization using Rosetta Resolver^®^ version 7.1 Affymetrix Rosetta-Intensity Profile Builder software (Rosetta Inpharmatics, Kirkland, WA). Statistically significant genes were identified by one-way ANOVA with a false discovery rate (Benjamini-Hochberg test) ≤ 0.01. A total of ~890 biosets were created and annotated as described below using information from the original study. All statistically filtered gene lists were uploaded into NextBio after filtering for ∣fold-change∣ ≥ 1.2. For analysis of effects of hypophysectomy and GH on liver STAT5b function, lists of statistically significant sets of genes were derived directly from the analysis in Wauthier et al. (2010) [36] (S1A1 Table; GSE17644). Only genes that exhibited a significant change (p-value < 0.005, the p-value cutoff used in this study) were used for the analysis. Fold-change values for the genes in each of the biosets in this study were uploaded to NextBio and compared to the STAT5b biomarker gene set.

### Annotation of a mouse liver gene expression compendium

All biosets were annotated for study characteristics allowing a systematic assessment of the effects of different factors on liver STAT5b function (described in detail in Oshida et al., 2015)[30]. Further details of each experiment not in our annotated compendium are available from the original submissions in GEO (http://www.ncbi.nlm.nih.gov/geo/) or ArrayExpress (http://www.ebi.ac.uk/arrayexpress/).

### Identification of STAT5b biomarker genes

Biomarker genes were identified as detailed in Oshida et al. (2015)[30] and were derived from NextBio biosets of statistically filtered genes. STAT5b biomarker genes are defined as those that 1) exhibited statistically significant changes between males and females in 4 or more of the 6 male liver vs female liver comparisons; 2) exhibited the same direction of change in each of the comparisons; 3) the average ∣fold-change∣ ≥ 1.5 across the comparisons; and 4) required STAT5b for differences in expression between male and female liver. The final STAT5b biomarker gene set was comprised of 144 genes (74 with increased expression and 70 with decreased expression) that exhibited similar regulation in male vs. female liver comparisons, all of which were dependent on STAT5b for sex-specific expression. The STAT5b biomarker was imported into NextBio without any further filtering. The list of STAT5b biomarker genes is found in Oshida et al. (2015)[30]. Based on our analysis procedures, each gene in the biomarker exhibited a robust, consistent response between sexes that was dependent on STAT5b. These procedures insured that the biomarker could accurately predict changes in liver STAT5b function (see below).

### Comparison of the STAT5b biomarker gene set to a microarray database

A rank-based nonparametric analysis strategy called the Running Fisher algorithm [37] and implemented within the NextBio database (http://www.nextbio.com/) environment was used for evaluating changes in liver STAT5b function. Details about the algorithm are discussed in Kupershmidt et al. (2010)[34]. The Running Fisher algorithm computes similarity p-values between two biosets by a Fisher exact test. This normalized ranking approach enables comparability across data from different studies, microarray platforms, and analysis methods by removing dependence on absolute values of fold-change, and by minimizing some of the effects of normalization methods used, while accounting for the level of genomic coverage by the different platforms. The STAT5b biomarker gene set was compared to each bioset in NextBio using the Running Fisher algorithm. The p-value of the similarity and the direction of correlation were exported, and after conversion of the p-values to–log(p-value) and negative correlations to negative numbers, the data was used to populate a master table containing information about each bioset. This final master table enabled the determination of effects on STAT5b function by broad categories of factors (e.g., chemical treatment) as well as individual factors (e.g., phenobarbital). This same strategy was employed to identify biosets that exhibited effects on AhR, CAR and PPARα. Predictions for factors that result in activation or suppression of these transcription factors [31–33] were included in the same master table to allow direct comparisons with effects on STAT5b in the same biosets.

### Classification prediction of STAT5b function

In this and in our previous studies [30–33], the biomarker gene sets were compared to known positives and negatives using the Running Fisher algorithm. Preliminary studies with the biomarkers for AhR, CAR and PPARα showed that a cutoff of Running Fisher algorithm p-value ≤ 10^−4^ (after a Benjamini Hochberg correction of α = 0.001) resulted in a balanced accuracy of 95%, 97%, and 98% for AhR, CAR and PPARα, respectively[31–33]. Applying the same test to the dataset using the STAT5b biomarker resulted in a cutoff of p-value = 2E-4, which for consistency with the previous studies was rounded to p-value = 10^−4^. Using a cutoff of 10^−4^ the balanced accuracy of STAT5b activation (masculinization) or STAT5b suppression (feminization) was 99% or 97%, respectively [30].

### Comparison of the expression of individual genes with STAT5b activation

Expression data for specific genes (i.e., *Pparg*, *Srebp1c*, *Srebp2*, *Lxra*, and *Lxrb*) was obtained from the NextBio database based on microarray comparisons. All expression values (fold-changes) for each gene were used to populate the compendium, allowing a direct comparison between changes in the expression of specific genes and STAT5b predictions. In making the comparisons, only biosets in which gene expression was altered were used in the comparisons, as values of “0” may also represent “no value” if the microarray platform did not query the gene or allow assessment of changes in that gene. To determine statistical significance of enrichment of gene expression changes with STAT5b functional status, statistical analyses were performed using a Fisher's exact test in EXSUS v8.0 (CAC EXICARE Corp., Japan). The numbers of biosets with increased or decreased expression of each transcription factor were compared to each other in bioset groups with STAT5b activity up or down based on a STAT5b unchanged bioset group.

### Additional computational analyses

Heat maps were generated using Eisen Lab Treeview software (http://rana.lbl.gov/EisenSoftware.htm).

## Results

To screen for hormones and chemicals that alter liver STAT5b function, we compared a biomarker for liver STAT5b-dependent gene expression to annotated biosets in a gene expression compendium using the fold-change rank-based nonparametric Running Fisher algorithm [34] as described in the companion publication [30]. These methods reliably predicted activation or suppression of liver STAT5b function, resulting in a balanced accuracy of 99% and 97%, respectively, and were used to identify genes, diets and infections that alter STAT5b[30]. In the present study, an identical screening strategy was used to identify hormones and chemicals that alter liver STAT5b function.

### Hormonal regulation of STAT5b function in mouse liver

**Fig 1:**
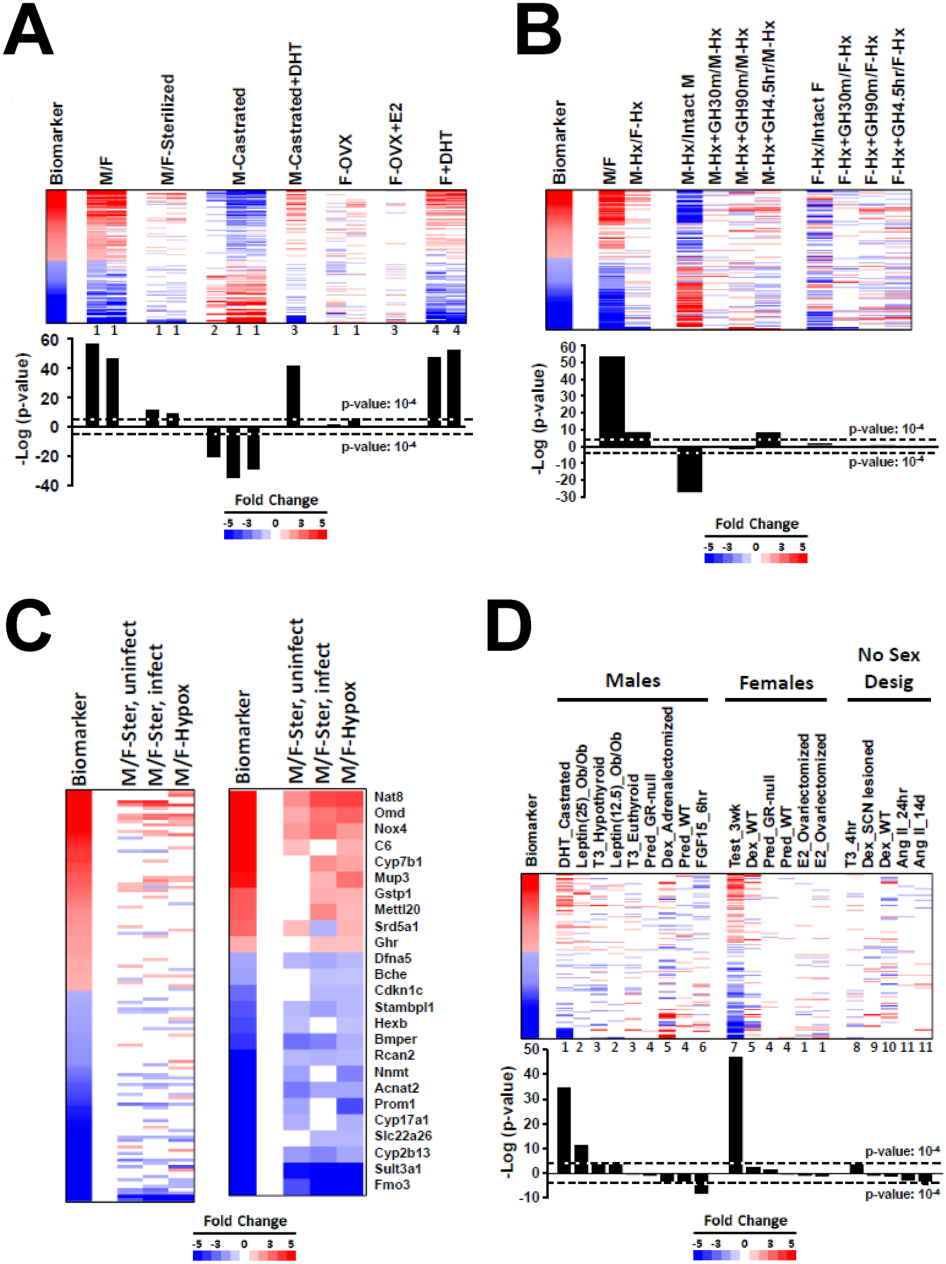
Hormonal modulation of liver STAT5b function. A. Activation of STAT5b function in testosterone-treated mouse liver. The STAT5b biomarker gene set was examined in the indicated biosets from GSE6632, GSE21065, and GSE13265. (Top) Expression behavior of the STAT5b biomarker gene set. The biomarker genes were rank-ordered based on fold-change. Numbers at the bottom of each lane refer to the following studies from which they were derived: 1, GSE21065; 2, GSE6632; 3, GSE13265; 4, GSE13388. (Bottom)–log(p-values) of the similarity to the STAT5b biomarker. M/F, male vs. female; M/F-sterilized, male vs. female comparison in untreated gonadectomized mice; M-castrated, castrated vs. intact mice; M-castrated+DHT, castrated mice treated with dihydrotestosterone (DHT; subcutaneous 5-mg DHT pellet, designed to be released over 21d and giving a plasma level of 1–2 ng DHT/ml vs. castrated mice treated with vehicle; F-OVX, ovariectomized vs. intact mice; F-OVX+E2, ovariectomized estradiol-treated (subcutaneous 0.5-mg E2 pellet (plasma level of 300 pg/ml) designed to be released over 21d) vs. ovariectomized vehicle-treated mice; F+DHT, intact female mice treated with DHT (subcutaneous injections of 100ul sesame oil containing 0.9mg testosterone twice a week for 3 weeks) vs. intact female mice treated with vehicle. B. Effects of pituitary GH modulation on liver STAT5b function. Biosets were derived from Wauthier et al. (2010) (GSE17644) and compared to the STAT5b biomarker gene set. Hypophysectomized (Hypox) male and female mice were given a single ip injection of rat GH (125 ng/g body weight) and killed 30 or 90 min later. Other Hypox male and Hypox female mice were given two ip injections of GH, spaced 4 h apart, and killed 30 min after the second GH injection. (Top) Expression behavior of the STAT5b biomarker genes. Genes were rank-ordered based on fold-change in the biomarker. (Bottom)–log(p-values) of the similarity to the STAT5b biomarker gene set. M/F, male vs female; M-Hx/F-Hx, Hypox males vs. Hypox females; M-Hx/Intact M, Hypox male vs. intact male; M-Hx+GH30m/M-Hx, Hypox males 30 min after a pulse of GH vs. Hypox males; M-Hx+GH90m/M-Hx, Hypox males 90 min after a pulse of GH vs. Hypox males; M-Hx+GH4.5h/M-Hx, Hypox males 4.5 hr after initial exposure to GH vs. Hypox males; F-Hx/Intact F, Hypox female vs. intact female; F-Hx+GH30m/F-Hx, Hypox females 30 min after a pulse of GH vs. Hypox females; F-Hx+GH90m/F-Hx, Hypox females 90 min after a pulse of GH vs. Hypox females; F-Hx+GH4.5h/F-Hx, Hypox females 4.5 hr after initial exposure to GH vs. Hypox females. C. Residual sex differences after sterilization and hypophysectomy. Two biosets from sterilized mice (one from uninfected mice and one from mice infected with *Coxiella burnetii*, GSE21065) were compared to a bioset from Hypox males vs. Hypox females (GSE17644 described above). (Left) Expression of all genes in the STAT5b biomarker gene set. (Right) Expression of genes that exhibited significant alteration in one or both of the M/F-sterilized biosets and the M-Hx/F-Hx bioset. D. Effects of hormone treatment on liver STAT5b function. Biosets derived from mice treated with the indicated hormones were separated into studies performed in male mice, female mice, and mice with no designation of sex. Numbers at the bottom of each lane refer to the specific studies from which each was derived: 1, GSE13265; 2, GSE19185; 3, GSE32444; 4, GSE21048; 5, GSE24256; 6, GSE29426; 7, GSE13388; 8, GSE21307; 9, GSE564; 10, GSE9630; 11, E-TIGR-12.

Many hormones and cytokines can affect the activity of STAT5b in various tissues [37–39], but a comprehensive screen to identify hormones that alter STAT5b function in liver cells has not been carried out. The effects of dihydrotestosterone (DHT) and estradiol (E2) were first examined either when given directly by injection, or indirectly, by examining the effects of hormone ablation by castration and ovariectomy (biosets derived from studies found in Gene Expression Omnibus (GEO): GSE21065, GSE13265, GSE13388, GSE6632). Male to female comparisons showed striking similarity based on expression of STAT5b biomarker genes (see heatmap) and significant correlation to the STAT5b biomarker gene set, as expected (Fig 1A, **M/F**). The significance of the correlation was greatly diminished in male vs. female comparisons examined 3 weeks after sterilization of both sexes (from GSE21065, one from uninfected mice and one from mice infected with *Coxiella burnetii*). Castration of males led to a reversal of the gene expression pattern; male-predominant genes decreased in expression and female-predominant genes were increased. This same pattern was observed in intact male STAT5b-null mice compared to intact male wild-type mice [30] and reflects the ability of STAT5b, when activated in the liver by the male, pulsatile plasma GH pattern, to not only activate male-specific genes but also to suppress female-specific genes [12], a pattern that is reversed in the absence of testosterone or when STAT5b is deleted. Consistent with these effects, the male pattern of gene expression was mostly restored in castrated male mice given DHT compared to untreated castrated males. The gene expression pattern did not change significantly following ovariectomy (F-OVX) or when ovariectomized female mice were treated with E2 (F-OVX+E2). In contrast, the male-like expression pattern of the STAT5b biomarker gene set was replicated in intact females given DHT. There were no biosets from male mice administered E2 or E2-like compounds in our compendium. Taken together, these comparisons are consistent with STAT5b playing an important role in determining the androgen-driven sexually dimorphic gene expression pattern in the mouse liver.

The impact of hypophysectomy (Hypox) and a physiological replacement dose of GH on the STAT5b biomarker gene set were examined using gene lists derived from a microarray study [36] (GSE17644). RNA was isolated from livers of adult mice that were: intact males and intact females; Hypox males and Hypox females; Hypox male and Hypox female mice treated with a single GH pulse and killed either 30 or 90 min later; and Hypox male and Hypox female mice treated with two GH injections, spaced 4 h apart, and killed 30 min after the second GH injection (4.5 hr after initial exposure). The bioset from intact male vs intact female mice showed extensive overlap with the STAT5b biomarker, as expected (Fig 1B). The bioset of Hypox males vs Hypox females showed a major decrease in the changes in the biomarker, but surprisingly, the significance was not completely abolished (c.f., -log(p-values) in Fig 1B). Like castration, Hypox in males (i.e., Hypox males vs. intact males) reversed the pattern of STAT5b biomarker genes, i.e., suppression of male-specific genes and activation of female-specific genes. Treatment of the Hypox males with GH for 4.5 hr but not 30 or 90 min GH treatment resulted in a partial recovery of masculinization. In contrast, neither Hypox in females (i.e., Hypox females vs. intact females) nor GH treatment of Hypox females had significant effects on liver STAT5b function. Overall, these results are consistent with known effects of GH on STAT5b activity in adult male mouse liver.

Given that there were significant residual sex differences 3 weeks after removal of sex organs (Fig 1A) and also a similar time after Hypox (Fig 1B) as assessed by our STAT5b biomarker, we investigated if there was a common set of genes that retained sex specificity between these biosets (Fig 1C, **left**). In general, the genes that retained sex differential expression were those biomarker genes that exhibited the greatest sex differences (e.g., see the cluster of down-regulated genes at the bottom of the biomarker). Genes were identified that exhibited significant alteration in one or both of the M/F-sterilized biosets and the M/F-Hypox bioset. Out of 144 genes in the biomarker, 25 genes (10 up-regulated and 15 down-regulated) exhibited consistent sex-specific expression after sterilization and after Hypox (Fig 1C, **right**). In all cases, the extent of the sex differences was greatly diminished compared to that in the biomarker, i.e., there is partial retention of sex specificity, indicating that testosterone and GH have predominant effects on their sex-specific expression. Conceivably, the residual sex specificity of these genes in the absence of sex hormones and in the absence of GH and other pituitary-regulated hormones may result from sex-dependent regulation by factors encoded on the X and Y chromosomes.

All hormone and cytokine treatments in the compendium were examined for effects on liver STAT5b function. The biosets from mice treated with the hormones discussed above were included for comparison. In mouse primary hepatocytes, many cytokine treatments (interferon-β, interleukin-17, interleukin-1β, interleukin-6, tumor necrosis factor-α, or a mixture of interleukin-3, interleukin-6, stem cell factor, thrombopoietin, and Flt3 ligand) had no significant effect on expression of the STAT5b biomarker gene set (data not shown). Biosets from treated mice were divided into males, females, and mice in studies where no sex designation was provided (Fig 1D). (For a list of the surprisingly large number of studies with no sex designation in either the GEO submission or in the Methods sections of the published papers, see Oshida et al. (2015) [30].)

In addition to the “re-masculinization” of castrated mice by dihydrotestosterone treatment described above (GSE13265), masculinization of a small subset of the STAT5b biomarker genes was observed in male hypothyroid but not male euthyroid mice given thyroid hormone (T3) treatment (1 mg/kg) for 24 hr (GSE32444) or in 15d old euthyroid mice of unknown sex given T3/T4 treatment (50 μg of T4 + 5 μg of T3 per 100 g body weight) for 4 hr (GSE21307). Consistent with these partial masculinizing effects of T3, treatment of hypothyroid male mice with GC-1 (1 mg/kg), a T3 mimetic, caused masculinization (p-value = 2.7E-6; GSE32444; data not shown). There is molecular evidence of direct interactions between STAT5b and thyroid hormone receptor-β 1 (TRβ1); however, in at least *in vitro* models, the interactions are mutually antagonistic [40, 41]. Other studies showed positive cooperation between T4 and GH-activated STAT5 in human skin fibroblast cultures [42]. T3 directly regulates Sertoli and Leydig cell functions by increasing Leydig cell luteinizing hormone (LH) receptor numbers and mRNA levels of steroidogenic enzymes and regulators, including steroidogenic acute regulatory protein (StAR). T3 stimulates basal and LH-induced secretion of progesterone, testosterone, and E2 by Leydig cells (reviewed in Maran, 2003[43]). T3 and T3 mimetics could thus act as masculinization factors by increasing production and secretion of testosterone levels.

Livers of male mice were partially feminized following treatment with the glucocorticoid receptor (GR) agonist prednisolone (1 mg/kg) for 2.5 hr in wild-type but not glucocorticoid receptor-null mice (GSE21048) (Fig 1D). Adrenalectomized male mice 6 hr after injection with another GR agonist (dexamethasone) (1 mg/kg) approached significance for feminization (p-value = 4E-4) (GSE24256). The molecular basis of this partial feminization is not known, but could involve two non-mutually exclusive mechanisms. First, feminization may occur through direct inhibition of STAT5b activity by activated GR. STAT5b and GR interact directly through the STAT5b N-terminus and the AF-1 domain of GR (reviewed in Henninghausen and Robinson, 2008 [44]). Dexamethasone-activated GR synergizes with prolactin-stimulated STAT5b to increase the expression of the β-casein gene in mammary epithelial cells [45]. Although the β-casein gene is a target of positive interactions between STAT5b and GR, we hypothesize that activated GR in some cell- or gene-specific contexts could inhibit STAT5b regulation of the biomarker genes. Second, feminization may occur via the down-regulation and decreased abundance of components of JAK-STAT5b signaling after exposure to GR agonists. In primary T-cells, dexamethasone inhibited IL-2-induction of STAT DNA binding, tyrosine phosphorylation, and nuclear translocation. The mechanism of inhibition involved suppression of *IL-2 receptor* and *Jak3* gene expression [46].

Feminization in male mice was also observed after treatment of wild-type mice with mouse fibroblast growth factor 15 (FGF15) (0.15 μg/g body weight) for 6 hr (GSE29426). Mouse FGF15 is secreted from the small intestine in response to feeding and has effects on energy homeostasis, indicated by increases in circulating levels lead to suppression of liver gluconeogenesis [47]. Fgf15-null mice have reduced liver glycogen storage and are glucose-intolerant [48]. Interactions between FGF15 and STAT5b have not been previously reported.

Angiotensin II treatment resulted in partial feminization (E-TIGR-12) in mice with no sex designation. Angiotensin is a peptide hormone in the renin-angiotensin system that signals via a G-protein coupled receptor causing vasoconstriction and a subsequent increase in blood pressure. Angiotensin II activates STAT5b in cardiac myocytes in a JAK2-dependent manner [49] and in vascular smooth muscle cells [50].

A number of points are worth considering when evaluating the effects of these hormonal treatments on liver STAT5b function. First, many of the treatments induce partial feminization or masculinization of the STAT5b biomarker gene set. Some of the treatments, e.g., the 2.5 hr and 6 hr treatments of prednisone and FGF15, respectively, may be too short to confer extensive feminization owing to the time required for decay of male-specific transcripts. Second, the context of the masculinization observed in our analysis is important, as the masculinization may actually represent “re-masculinization” of a liver feminized by another factor. An example of this “re-masculinization” is described above when castrated mice were given DHT. Another example is described in the next section.

### Feminization in obesity and diabetes models is corrected by leptin and resveratrol

**Fig 2:**
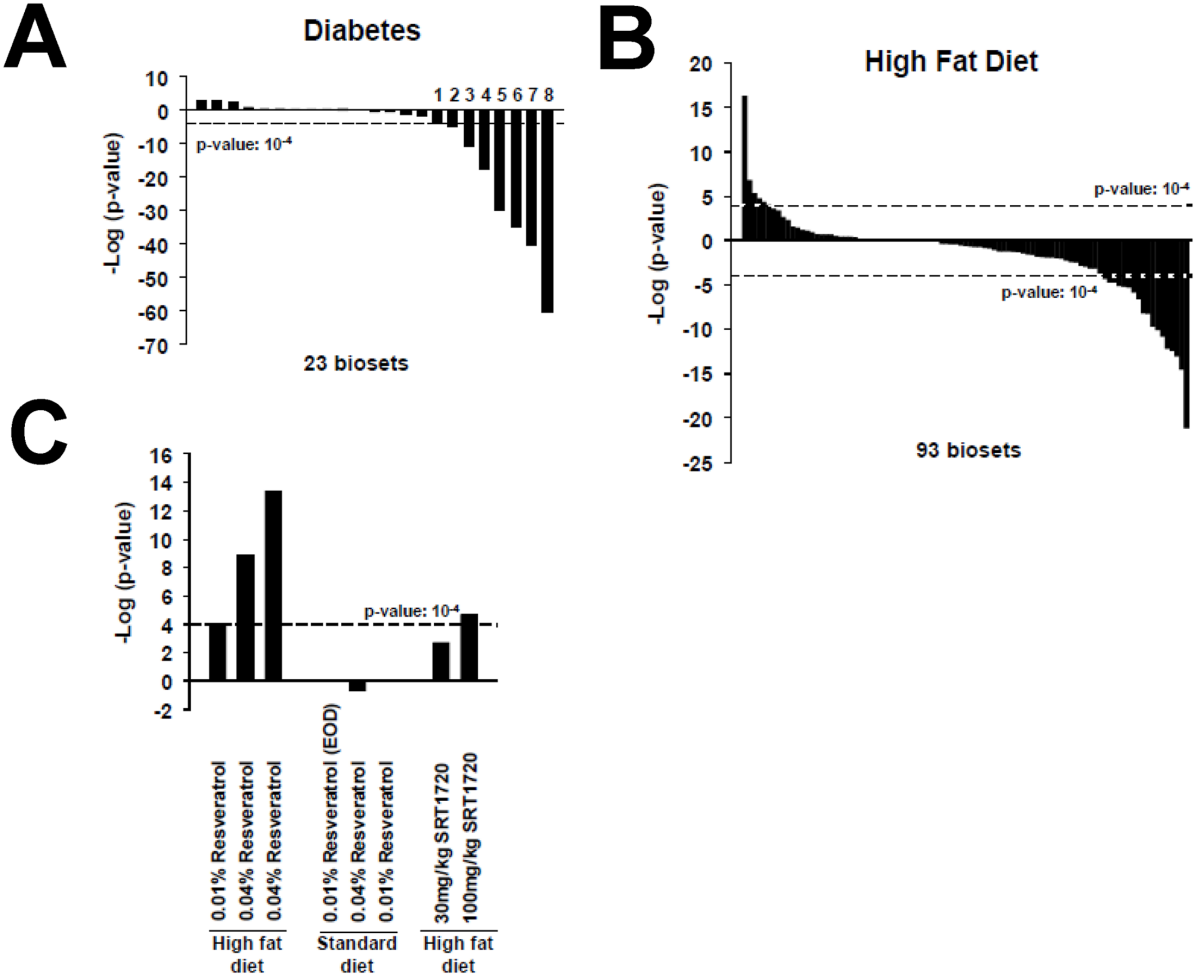
Feminization of the liver in models of obesity and diabetes and “re-masculinization” by resveratrol. A. Feminization in models of diabetes. The biosets which caused feminization (suppression of STAT5b function) were from the following studies: 1) GSE30140; 2) GSE30140; 3) GSE10785; 4) GSE30140; 5) GSE10785; 6) GSE38067; 7) GSE10785; 8) GSE10785. B. Effects of high fat diets on STAT5b. The effects of high fat diet on STAT5b function are based on an analysis of 93 comparisons. C. “Re-masculinization” by resveratrol in mice fed a high fat diet but not a standard diet. Three biosets show masculinization after feeding mice a high fat diet with resveratrol vs. high fat diets alone (from GSE11845, GSE6089). Three biosets from mice fed a standard diet plus resveratrol vs. a standard diet alone did not show significant effects (from GSE11845). One of the two biosets from mice fed a high fat diet with SRT1720 vs. high fat diet alone (from GSE19102) exhibited partial “re-masculinization”. EOD, every other day.

Masculinization was observed after leptin treatment (12.5 or 25 ng/hr) of adult leptin-defective ob/ob mice for 12d (GSE19185) (Fig 1D). There is evidence that leptin *per se* does not cause masculinization but rather causes “re-masculinization” of the feminization in the leptin-deficient *ob/ob* mice, which are obese and diabetic. This “re-masculinization” of the feminized liver hypothesis is consistent with a number of observations. First, obese and/or diabetic mice, in which there are metabolic derangements, showed consistent feminization. Out of 23 biosets, 8 biosets from three studies showed feminization when obese or diabetic mice were compared to their lean or non-diabetic counterparts, whereas no biosets showed significant masculinization (Fig 2A). In 93 biosets from mice fed a high fat diet, more biosets showed feminization (18) than masculinization (6) (Fig 2B). Overall, high fat diets, obesity, metabolic syndrome, and diabetes were consistently associated with feminization of liver STAT5b function. These effects are reminiscent of livers where the *Stat5a/Stat5b* locus is deleted, which results in hepatosteatosis, glucose intolerance, insulin resistance, and obesity [17, 51].

Second, leptin corrects the defect in *ob/ob* mice that leads to feminization. Leptin at a dose of 12.5 ng/hr significantly lowered blood glucose while a dose of 25 ng/hr normalized plasma glucose and insulin without significantly reducing body weight, establishing that leptin exerts its most potent effects on glucose metabolism (Hedbacker et al., (2010) [52] (GSE19185)) in parallel with the masculinization seen in Fig 1D. Leptin can activate STAT5b in rat liver [53]. Further, leptin treatment in rat pancreatic beta cells leads to JAK2-dependent phosphorylation of STAT5b as well as STAT5b nuclear translocation and DNA binding [54]. In the hypothalamic arcuate nucleus in mice, leptin stimulates STAT5 phosphorylation [55]. Similar to the effects of leptin, we found that treatment with resveratrol or the resveratrol derivative SRT1720, both of which have normalizing effects on glucose levels [56, 57], also caused masculinization of diabetic mice on a high fat diet but not mice on a standard diet that were not diabetic (Fig 2C). Taken together, diabetes associated with obesity caused feminization, while correction of the diabetes defect by leptin or resveratrol treatment caused re-masculinization. Although the mechanisms by which leptin and resveratrol cause re-masculinization are unknown, it is interesting to note that leptin acts at all levels of the hypothalamus-pituitary-gonadal (HPG) axis in males [58] and glucose negatively regulates GH levels [6, 59].

### Effects of chemical exposure on liver STAT5b function

**Fig 3:**
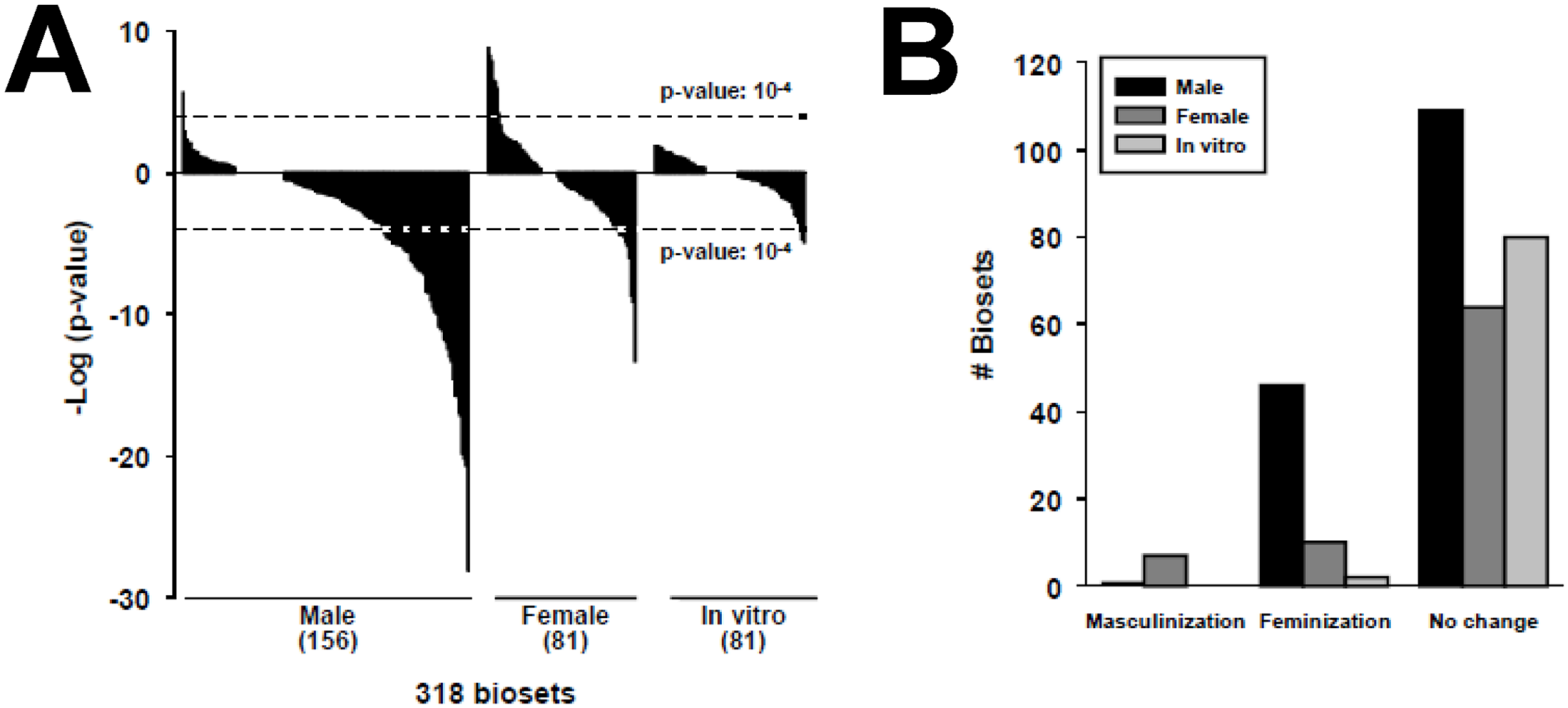
Effects of chemical exposure on STAT5b. A. Distribution of chemical effects on STAT5b biomarker activation or suppression in males and females in vivo or in in vitro experiments. The p-value cutoffs for similarity to the STAT5b biomarker for the chemical comparisons are shown. B. Number of biosets that exhibited masculinization or feminization of the liver transcriptome after chemical exposure in male and female mice and in in vitro experiments.

The STAT5b biomarker gene set was compared to 318 chemical vs. control biosets representing exposure to 156 chemicals. The distributions of the p-values for 156 biosets in males, 81 biosets in females, and 81 biosets from *in vitro* studies are shown in Fig 3A and 3B. The most frequent effect of chemical exposure was feminization in males. In males, ~29% of the biosets were feminized, whereas only one bioset (GC-1 treatment, discussed above) was masculinized. In females, ~9% of the biosets were masculinized and ~12% were feminized. Masculinization of the female liver transcriptome was observed after exposure to benzofuran, coumarin, and methylene chloride in a 90-day exposure study [60]. In general, the effects of chemical exposure on feminization were more significant in males (p-values ≥ 10^−27^) than effects of feminization (p-values ≥ 10^−13^) or masculinization (p-values ≥ 10^−8^) in females. Because *in vitro* cultures often lack the ability to respond normally to the male-dependent pattern of pulsatile GH secretion [61], *in vitro* cultures would not be expected to serve as a suitable model for assessing the impact of chemical exposure on liver STAT5b function. Indeed, of the 82 biosets from *in vitro* cultures encompassing exposure to 38 chemicals, only 2% reached significance (0 masculinized and 2 feminized). The significance of these correlations (p-values ≥ 10^−4^) was far lower than the range of significance values for the *in vivo* comparisons. Chemicals that suppressed STAT5b function in vivo but not in vitro included TCDD, benzo[a]pyrene, dimethylbenzanthracene, lipopolysaccharide, phenobarbital, quercetin, and WY-14,643.

### Most chemicals that caused feminization also activated xenobiotic-responsive receptors

**Fig 4:**
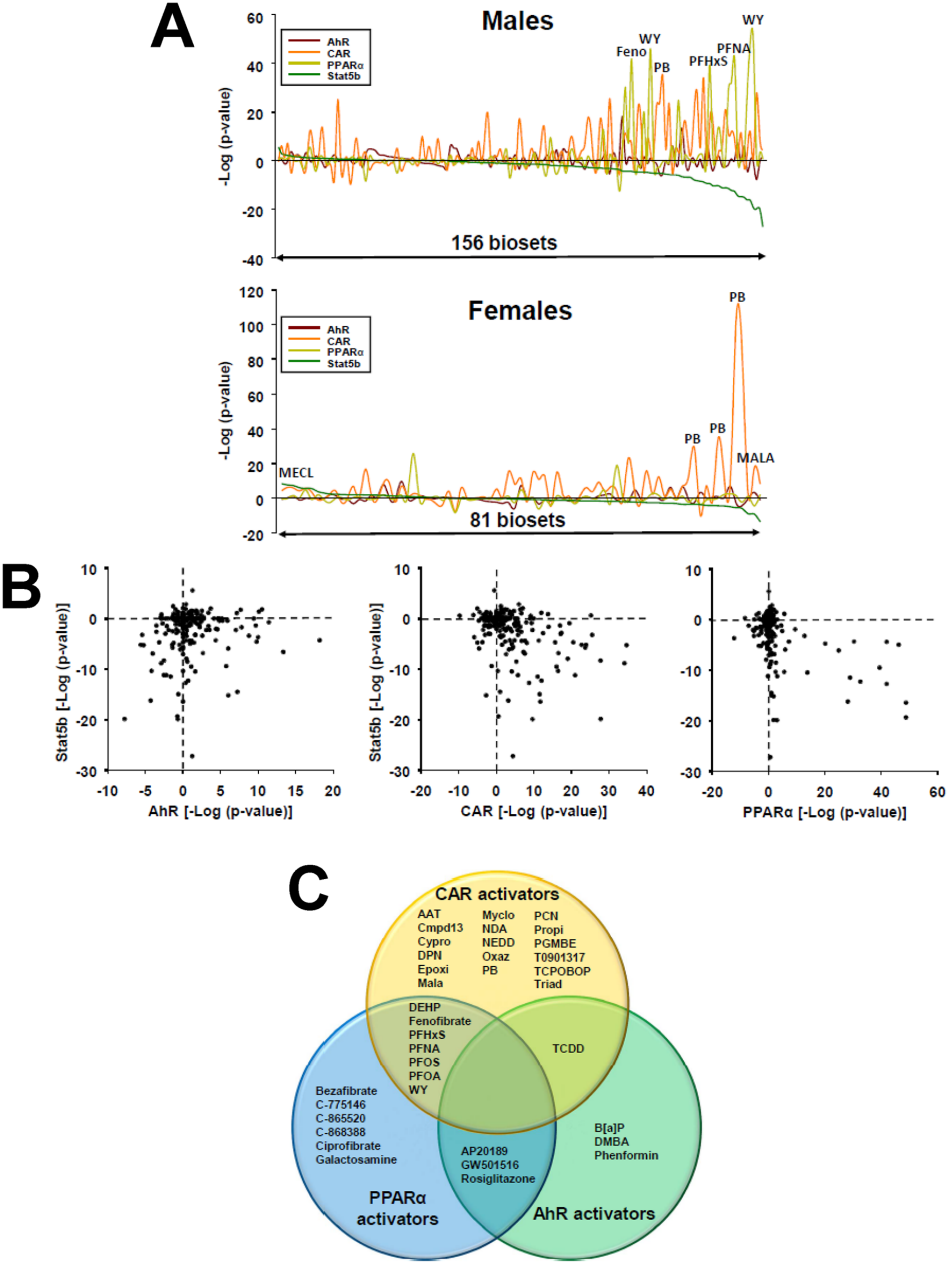
Suppression of liver STAT5b function (liver feminization) by activators of xenobiotic receptors. A. Comparison STAT5b, AhR, CAR and PPARα modulation in male and female mice. The STAT5b biomarker gene set-derived–log(p-value)s from the Running Fisher tests were rank ordered and then compared to the predictions of chemically-treated male mice (top) or female mice (bottom) for the biomarker gene sets for AhR, CAR or PPARα, which were derived using similar methods [31–33]. Biosets with the most significant masculinization are on the left; biosets with most significant feminization are on the right. A number of chemicals that significantly alter STAT5b and a xenobiotic receptor are shown. Abbreviations are found in Fig 4C, legend. B. Relationships between activation of AhR, CAR, or PPARα and modulation of STAT5b in male mice. The–log(p-value)s for prediction of AhR, CAR or PPARα modulation (x-axis) vs. STAT5b modulation (y-axis) are shown. C. Chemicals that feminize the liver transcriptome in intact male mice. The Venn diagram shows those chemicals that cause liver feminization and activation of AhR, CAR and/or PPARα. Chemicals in the overlap regions are those that activate two of the three receptors. Abbreviations: AAT: aminoazotoluene; B[a]P: benzo[a]pyrene; Cmpd13: compound 13; Cypro: cyproconazole; DEHP: di(2-ethylhexyl) phthalate; DMBA: dimethylbenzanthracene; DPN: 2,3-bis(4-hydroxyphenyl)propionitrile; Epoxi: epoxiconazole; MALA: malathion; MECL, methylene chloride; Myclo: myclobutanil; NDA: 1,5-naphthalenediamine; NEDD: n-(1-naphthyl)ethylenediamine dihydrochloride; Oxaz: oxazepam; PB: phenobarbital; PCN: pregnenolone-16 alpha-carbonitrile; PFHxS: perfluorohexanesulfonic acid; PFNA: perfluorononanoic acid; PFOA: perfluorooctanoic acid; PFOS: perfluorooctane sulfonate; PGMBE: propylene glycol mono-t-butyl ether; Propi: propiconazole; TCDD: 2,3,7,8-tetrachlorodibenzo-p-dioxin; Triad: triadimefon; WY: WY-14,643. None of the chemicals activated all three receptors.

Preliminary examination of the chemicals that caused feminization indicated that many are reference chemical activators for the xenochemical receptors AhR, CAR or PPARα. To systematically determine the relationships between activation of these receptors and feminization in intact mice, previous predictions of receptor activation derived by biomarker-based approaches were used [31–33]. The predictions of each chemical comparison for hepatic STAT5b activation or suppression in livers of male or female mice were rank-ordered by–log(p-value) and compared to predictions of AhR, CAR and PPARα activation or suppression. Fig 4A (**top, far right**) shows that many of the treatments that feminized the livers of male mice were associated with significant activation of CAR or PPARα, with fewer examples for activators of AhR. A smaller number of biosets from chemical-treated female mice resulted in feminization/suppression of STAT5b function coincident with activation of CAR (Fig 4A, **bottom, far right**).

The co-occurrence of feminization and xenobiotic receptor activation was a frequent event. Out of the 46 biosets that were feminized by chemical exposure in males, or the 10 biosets feminized in females, 93% of the biosets in males and 80% of the biosets in females exhibited activation of AhR, CAR, or PPARα (Fig 4A). Plots of the relationships between predicted–log(p-value)s for the 156 biosets for activation or suppression of STAT5b function vs. activation or suppression of the three receptors in male liver show that the chemical treatments with the most significant activation of CAR and PPARα are the most likely to feminize the liver (Fig 4B, **bottom right quadrants**). Many of the chemical treatments that had effects on AhR also caused feminization, but the biosets included both those that suppressed as well as activated AhR. The specific chemicals that induced feminization in livers of male mice and activated one or more of the xenobiotic receptors are shown in Fig 4C. Several of these chemicals activated two of the three receptors. A smaller number of chemicals caused feminization with co-suppression of AhR activity (phenobarbital, bezafibrate, pregnenolone-16 alpha-carbonitrile, myclobutanil) or co-suppression of CAR activity (WY-14,643) (not shown). However, all of these treatments activated one of the other two receptors. Chemicals that feminized the liver in male mice but did not activate or suppress any of the three receptors included 24-norursodeoxycholic acid, galactosamine, and sebacic acid. The list of chemicals that modulate STAT5b and xenobiotic-activated transcription factors is found in S1 Table.

### Chemical and hormone exposure conditions that modulate STAT5b

**Fig 5:**
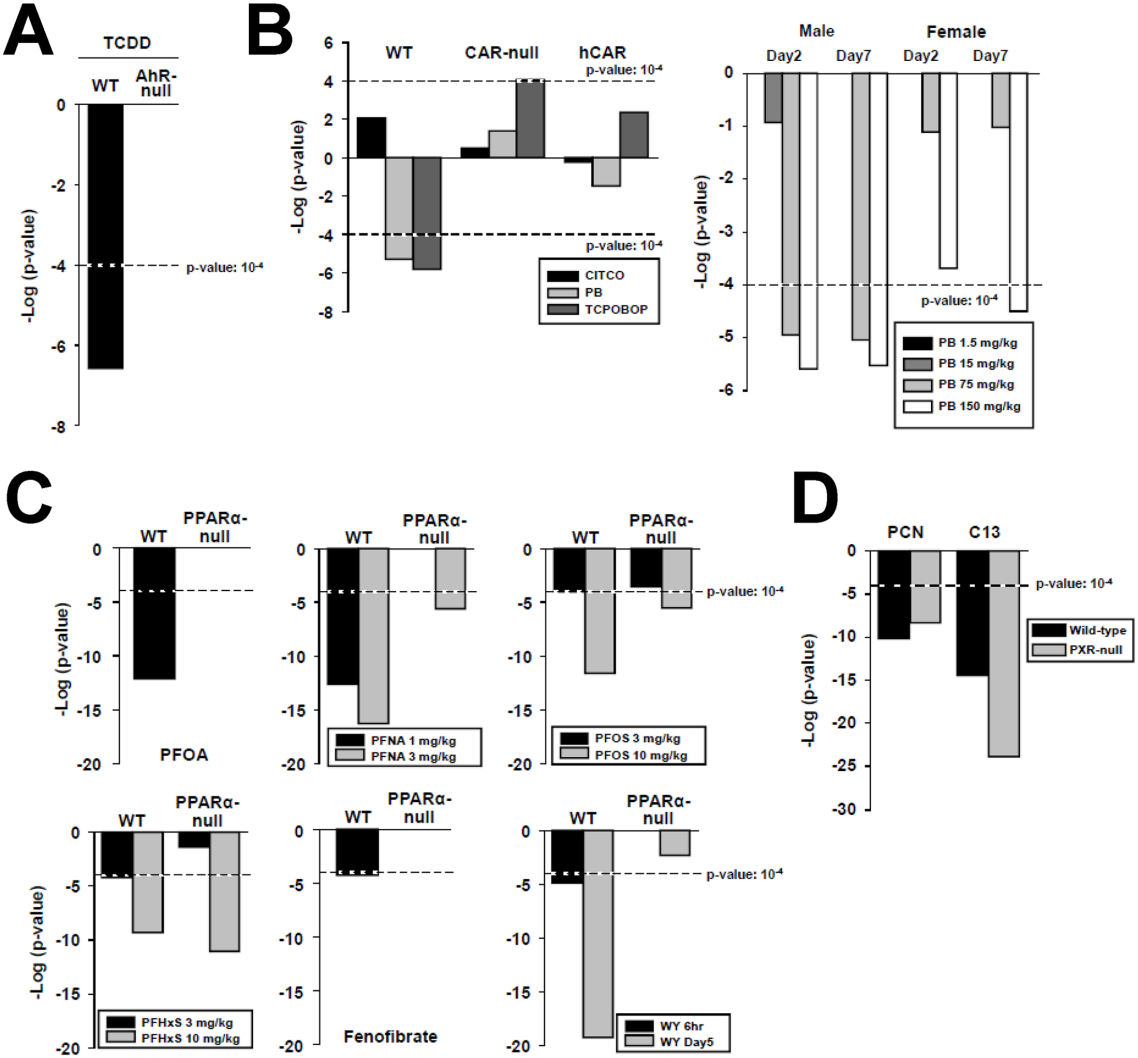
Dose- and receptor-dependence of feminization of the liver by chemical exposure. A. Feminization by TCDD is dependent on AhR. TCDD (1 mg/kg) was administered to wild-type or AhR-null mice for 19 hr (GSE15859). B. (Left) Feminization by phenobarbital and TCPOBOP is dependent on CAR. CITCO (30 mg/kg/day), phenobarbital (100 mg/kg/day) or TCPOBOP (3 mg/kg/once) were given to wild-type, CAR-null, or CAR-null mice expressing a human CAR (humanized CAR mice) for 3 days (GSE40120). (Right) Dose-dependent feminization by phenobarbital. Phenobarbital was given to male or female mice for 2 or 7 days at the indicated dose levels (from GSE54597). C. Feminization by structurally diverse PPARα activators. The indicated PPARα activators were administered to wild-type or PPARα-null mice from GSE55756 (PFHxS (10 mg/kg/day), PFNA (3 mg/kg/day) for 7 days), GSE9786 (PFOA (3 mg/kg/day) for 7 days), GSE22871 (PFOS (10 mg/kg/day) for 7 days), GSE8396 (fenofibrate (400 μl of 10 mg/ml), WY (400 μl of 10 mg/ml), 6 hr), and GSE8295 (WY (0.1% w/w) mixed in the food), 5 days). D. Feminization by two PXR activators is PXR-independent. Wild-type and PXR-null mice were exposed to PCN (GSE55746) (400 mg/kg/day for 4 days) or Compound 13 (C13, GSE23780) (150 mg/kg/day for 4 days).

To gain insights into the mechanism of liver feminization, activators of AhR, CAR, and PPARα were examined to determine if their effects on STAT5b function are receptor-dependent. In addition, two compounds that activate the xenobiotic receptor PXR were examined. TCDD caused feminization in wild-type but not AhR-null mice (from GSE15859) (Fig 5A). Similarly, the CAR activators phenobarbital and TCPOBOP caused feminization in wild-type but not CAR-null female mice, in which statistically marginal masculinization was observed in the case of TCPOBOP treatment (from GSE40120) (Fig 5B, **left**). No significant effects were observed in similarly treated CAR-null mice expressing a human CAR gene, even when using the CAR human-specific activator CITCO. In a separate study, phenobarbital treatment of male mice for 2 or 7 days caused feminization at the two highest doses tested, whereas in female mice, feminization was only seen at the highest phenobarbital dose at 7 days (from GSE54597) (Fig 5B, **right**). This sex difference suggests a greater sensitivity of male mice to the effects of phenobarbital on STAT5b-regulated genes. However, in the Geter et al. (2014) [62] study from which the microarray data originated, no apparent strong sex bias for gene expression was noted.

Six activators of PPARα were examined for effects on STAT5b function in wild-type and PPARα-null mice. Four perfluorinated compounds (PFNA, PFHxS, PFOS, PFOA) consistently caused feminization in wild-type mice (Fig 5C). Feminization was dependent on PPARα for PFOA and for PFNA at the low dose tested, as seen by comparing effects in PPARα-null mice. Feminization was markedly reduced in significance but not abolished for PFNA and for PFOS at the highest doses tested. At the highest dose tested PFHxS caused approximate equally significant feminization in both wild-type and PPARα-null mice, evidencing effects that are independent of PPARα. The hypolipidemic agents fenofibrate and WY-14,643 caused feminization in wild-type but not PPARα-null mice that increased in significance with time in the case of WY-14,643 (Fig 5C). Lastly, the effect of exposure to two PXR activators on STAT5b was examined in wild-type and PXR-null mice. Both PXR activators (PCN and “compound 13”) caused feminization of wild-type mice (Fig 5D; data from GSE23780 and GSE55746). The significance of the feminization was approximately the same (PCN) or increased (compound 13) in PXR-null mice, indicating feminization proceeds by a PXR-independent mechanism. Overall, it can be concluded that many of the chemicals that induce feminization (TCDD, phenobarbital, TCPOBOP, PFOA, fenofibrate, WY) require the xenobiotic receptor. However, the effects of other chemicals were independent of either PPARα (PFNA, PFHxS, PFOS) or PXR (PCN, Compound 13). As PFNA, PFHxS, PFOS, PCN, and Compound 13 activate CAR [31] (Fig 4C), we hypothesize that feminization is mediated by multiple receptor-dependent mechanisms, including one involving CAR.

Although the ability of activators of CAR and AhR to feminize the liver appears to be novel, there is evidence from previous studies that PPARα activators have effects on GH-regulated genes in the liver. Early studies of liver gene expression in rats exposed to PPARα activators showed altered regulation of classical target genes of STAT5b, including *CYP2C11*, *CYP2C12* [63], and *CYP2C7* [64]. PPARα- and GH-regulated pathways, including STAT5b activation, were found to be mutually antagonistic [65]. Consistent with this mutual antagonism, a number of targets of PPARα were found to be expressed at higher levels in the livers of STAT5b-null mice [66] as well as dwarf mice with mutations in *Prop1*, *Pit1* and *Ghrhr* genes [67], in which the liver gene expression pattern was strongly feminized compared to wild-type mice [30]. Overall, the hypothesis that chemical activators of PPARα lead to alteration of hormone-induced, STAT5b-regulated gene expression in tissues such as liver [65] is supported by the current study.

### Feminization is associated with increased expression of PPARγ

**Fig 6:**
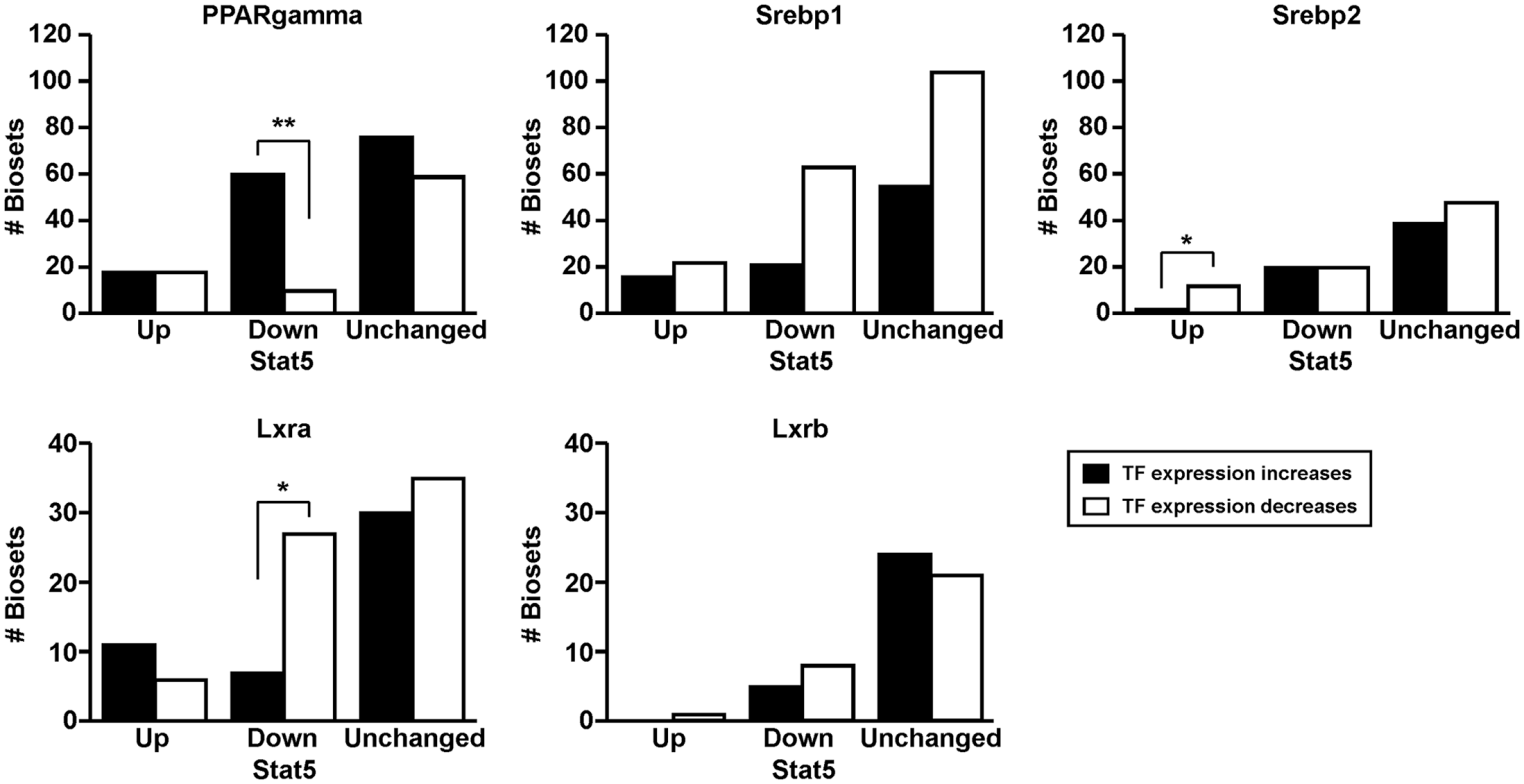
Relationships between expression of lipogenic transcriptional regulators and liver STAT5b activity status. Expression of the genes encoding the indicated transcription factors involved in lipogenesis was compared to the predictions of effects on liver STAT5b function using the STAT5b biomarker gene set. Increase or decrease in gene expression was defined as ≥ 1.5-fold or ≤ -1.5-fold, respectively. Significant differences based on a Fishers exact test are indicated: p-values ≤ 0.05 are indicated with * and p-values ≤ 0.01 are indicated with **.

The increase in steatosis seen in STAT5b-null mice is hypothesized to occur through the activation of transcription factors that regulate triglyceride or cholesterol homeostasis [26]. Our computational approach allows a weight of evidence approach to identify consistent relationships between STAT5b activation/repression and expression of any gene in the database, including transcription factors, based on an analysis of large numbers of independent studies. Relationships were examined between STAT5b activity status and the expression of lipogenic transcriptional regulators that were previously investigated in STAT5a/b-null and STAT5b-null mouse studies and thought to provide key links between perturbations of STAT5b function and increases in liver steatosis, notably *Pparg*, *Srebp1*, *Srebp2*, *Lxra*, and *Lxrb*. Biosets were identified in which the transcription factor gene exhibited statistically significant changes in expression (|fold-change| ≥ 1.5). For biosets in which masculinization of liver STAT5b function occurred (i.e., STAT5b up), approximately equal numbers of biosets exhibited increases in *Pparg* expression as decreases (Fig 6). In contrast, 87% of the biosets (60 out of 69) that exhibited feminization of STAT5b function (STAT5b down) also exhibited increases in *Pparg* expression. Greater numbers of biosets exhibited decreases compared to increases in the expression of *Srebp1* and *Lxra* in biosets that exhibited feminization which was statistically significant for *Lxra*. In biosets which exhibited masculinization of STAT5b function, a greater number of biosets exhibited decreases in *Srebp2* expression than increases in expression. There was no apparent relationship between alteration in the expression of *Lxrb* and STAT5b status. In summary, 1) factors (biosets) associated with increased STAT5b activity/masculinization tend to decrease *Srebp2* expression, and 2) factors (biosets) associated with decreased STAT5b activity/feminization tend to increase *Pparg* and decrease *Lxra and Srebp1*. The relationship between feminization and increased expression of *Pparg* may provide a plausible explanation for increased steatosis in biosets in which there are decreases in STAT5b activity, similar to increases in steatosis in mice that lack a functional STAT5b [26].

## Discussion

**Fig 7:**
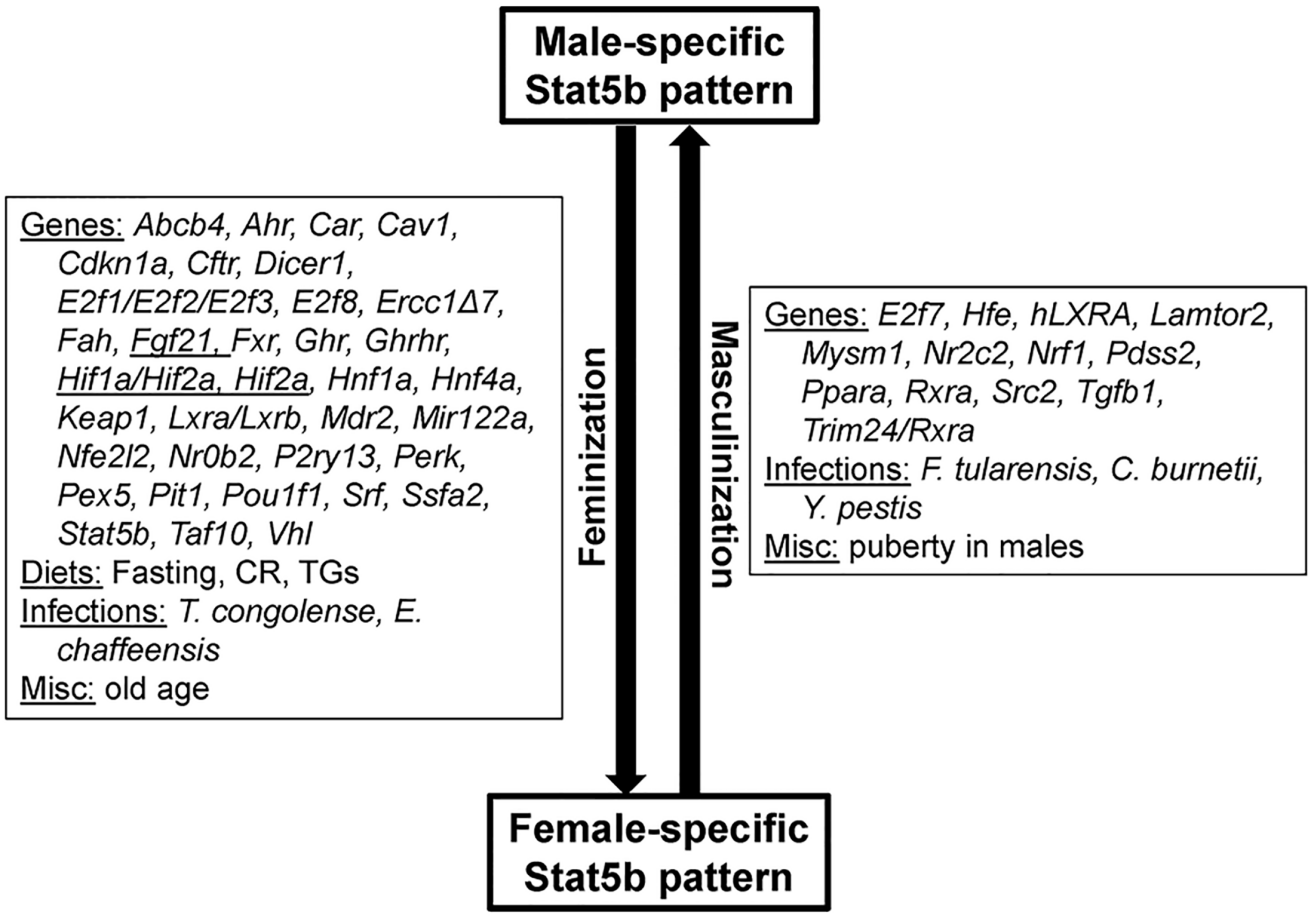
Summary of factors identified in this study that masculinize or feminize the liver transcriptome. Not all high fat diets caused feminization indicated as a parentheses around “high fat”. Abbreviations: BFUR, benzofuran; COUM, coumarin; MECL, methylene chloride; DHT, dihydrotestosterone; GH, growth hormone; T3, thyroid hormone.

We used a STAT5b biomarker gene set coupled with a rank-based statistical algorithm for similarity (Running Fisher algorithm) to identify hormones and chemicals that activate (masculinize) or suppress (feminize) STAT5b function in a mouse liver compendium (Fig 7**)**. This computational approach predicts changes in STAT5b activation status with a balanced accuracy for activation and suppression 99% and 97%, respectively, and identifies a large number of genes, diets, and infections that affect liver STAT5b function, as shown in the accompanying study [30]. Here, we observed an increase in liver STAT5b function (masculinization of liver gene expression) induced by exposure to DHT in either castrated mice or intact female mice, as well as a substantial loss of liver STAT5b function (loss of sex-differences) following hypophysectomy, which ablates pituitary GH and all other pituitary-derived hormones. We also discovered novel effects of T3 and leptin leading to masculinization, and of glucocorticoid receptor agonists and angiotensin II leading to feminization of liver STAT5 function; these findings warrant future studies to determine the molecular basis of these effects.

An important conclusion from our analysis is that chemical-induced loss of liver STAT5b function (feminization) is a relatively frequent phenomenon. Out of the 156 biosets from chemically-treated male mice, 29% showed significant suppression of liver STAT5b function/feminization. Only one chemical was found to masculinize the male liver (the thyroid hormone receptor agonist, GC-1). Further, 93% of the biosets that exhibited feminization of male liver were also associated with activation of one or more xenobiotic-responsive receptors, most commonly CAR or PPARα, and less frequently AhR. Given the relationship between suppression of STAT5b function and liver steatosis (see Introduction), it is conceivable that many of the chemical treatments examined in these biosets lead to steatosis. In fact, a number of chemicals that activate one or more of the above xenobiotic receptors perturb lipid homeostasis and cause steatosis. These chemicals include the AhR agonist ligand TCDD [68, 69] and perfluorinated chemicals [70, 71]. In our study, a large number of biosets encompassing both chemical-dependent and chemical-independent factors (e.g., hormones, diets, etc.) were evaluated for identification of consistent relationships between feminization of liver STAT5b function and alterations of lipogenic transcription factors. Increased expression of *Pparg* but not other lipogenic transcription factors was commonly associated with feminization (Fig 6). Eighty seven percent of the biosets that exhibited feminization of STAT5b function also exhibited increases in *Pparg* expression. In contrast, feminization was associated with decreases in the expression of *Srebp1* and *Lxra*. Further studies could test the hypothesis that in chemically-induced steatosis, there is a mechanistic link between suppression of STAT5b and increases in the expression of *Pparg* and regulated genes.

Chemical-induced feminization of the liver could occur by disruption of one or more sensitive nodes along the HPL-GH axis. In one model, mutual antagonism could come about directly in the liver through physical interactions between STAT5b and xenobiotic-activated receptors/transcription factors, or alternatively, by an indirect mechanism, such as competition for shared co-activator proteins. Circumstantial evidence for antagonism comes from observations of the behavior of genes co-regulated by PPARα and STAT5b, in which the absence of one transcription factor leads to increased expression of genes regulated by the other [65, 67]. In a second model, feminization could occur through alterations in circulating hormones that determine the pituitary GH secretory pattern. Treatment of male mice or rats with E2 was shown to antagonize hepatic expression of male-specific CYPs [72] and when administered to neonatal castrated rats can induce female-specific liver enzymes [73]. For example, DEHP, albeit at high doses, increased E2 levels [74] and caused feminization. However, to our knowledge, a transcriptome-wide study that evaluates liver effects of E2 treatment in male mice, which could be used to evaluate E2 effects on STAT5b, is lacking. Feminization may also occur through decreases in testosterone levels as was observed after castration (Fig 1A). CAR regulates a number of *Cyp* genes that metabolize testosterone to hydroxylated metabolites [75]. Serum levels of testosterone in male CAR-null mice are 2.5-fold higher than in wild-type mice, coincident with decreases in constitutive *Cyp2b9* and *Cyp2b10* in the liver [76]. Lastly, feminization could occur through increases in circulating glucose or triglycerides, both of which are elevated by GH and act to negatively regulate GH secretion [6, 59]. In models of diabetes and obesity, where circulating glucose levels are elevated, we consistently observed feminization of liver STAT5b function (Fig 2A), and feminization was at least partially reversed by leptin or resveratrol treatment (Figs 1D and 2C). Leptin was recently shown to normalize glucose levels in a number of rat models of diabetes (summarized in Greenhill, 2014[77]). Additionally, short-term exposure to GR agonists, which in our study caused feminization, increases glucose levels in part through stimulation of gluconeogenesis. Thus, we speculate that in some cases chemical exposure results in higher circulating glucose and fatty acids that can feedback to inhibit GH secretion at the hypothalamo-pituitary axis. This mechanism might not be relevant for PPARα activators that decrease circulating triglyceride levels [78] or for CAR activators that suppress glucose levels [79].

As the toxicology community continues to implement the recommendations of the NRC report on toxicity testing (NRC, 2007), the success of this endeavor will depend on the identification of pathways that cannot be evaluated using *in vitro* high throughput testing assays but are amenable to computational analysis, including the approaches described here using large and ever-increasing databases of gene expression data from chemical-treated liver and other tissues. In the present study and in the companion paper, we showed that hepatic STAT5b function is often altered in intact animals, especially in males, where feminization after genetic or chemical perturbation was often seen. In contrast, our evaluation of changes in 82 biosets from chemically-treated primary hepatocyte cultures, including chemicals that feminize the liver *in vivo*, showed that STAT5b function is rarely altered *in vitro*, and when altered, the changes are marginally significant compared to those seen in *in vivo* comparisons. In a tiered toxicity testing strategy in which chemicals are prioritized for further testing, a tier 1 *in vitro* screen would not identify those chemicals that perturb the HPL-GH axis and have an effect on STAT5b. In a prioritization strategy that identifies potential endocrine disruptors using *in vitro* screens, these chemicals would be ranked a lower priority for further testing. Only a tier 2 screen using short-term animal tests would identify chemicals that perturb the HPL-GH axis. As the toxicology community increasingly relies on *in vitro* tests for making decisions about chemical safety, a comprehensive tiered testing strategy is needed that identifies endocrine disrupting chemicals that perturb hormonal axes such as the hypothalamo-pituitary-liver GH axis.

## Supporting Information

S1 Table Chemical and hormone exposure conditions that modulate STAT5b. (XLSX)

## Abbreviations

CAR: constitutive activated receptor
CITCO: 6-(4-Chlorophenyl)imidazo[2,1-b][1,3]thiazole-5-carbaldehyde-O-(3,4-dichlorobenzyl)oxime
DEHP: di-(2-ethylhexyl)phthalate
DHT: dihydrotestosterone
E2: estradiol
FGF15: fibroblast growth factor 15
HPL: hypothalamic-pituitary-liver
PB: phenobarbital
PCN: pregnenolone-16-α-carbonitrile
PFHxS: perfluorohexane sulfonate
PFNA: perfluorononanoic acid
PFOA: perfluorooctanoic acid
PFOS: perfluorooctane sulfonate
PPAR: peroxisome proliferator-activated receptor
PPC: peroxisome proliferator chemical
PXR: pregnane X receptor
STAT5b: signal transducer and activator of transcription 5b
TCPOBOP: 1,4-Bis[2-(3,5-dichloropyridyloxy)] benzene
WY: WY-14,643
