# Correction: Chemical and Hormonal Effects on STAT5b- Dependent Sexual Dimorphism of the Liver Transcriptome

**DOI:** 10.1371/journal.pone.0161519

**Published:** 2016-08-16

**Authors:** Keiyu Oshida, David J. Waxman, J. Christopher Corton

The incorrect image is used in Fig 7. Please see the correct Fig 7 here.

**Fig 7:**
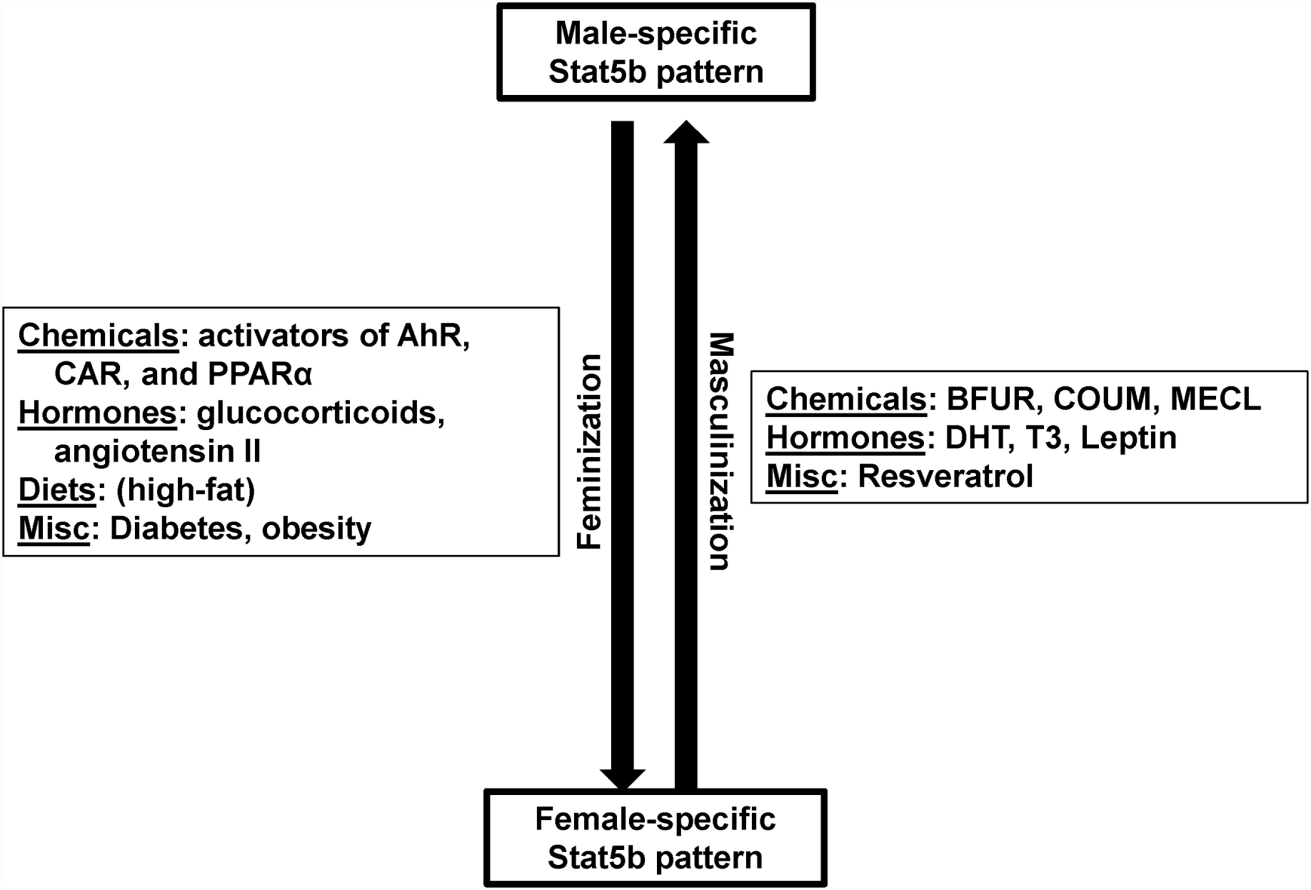
Summary of factors identified in this study that masculinize or feminize the liver transcriptome. Not all high fat diets caused feminization indicated as a parentheses around “high fat”. Abbreviations: BFUR, benzofuran; COUM, coumarin; MECL, methylene chloride; DHT, dihydrotestosterone; GH, growth hormone; T3, thyroid hormone.
